# To freeze or not to freeze: A culture-sensitive motion capture approach to detecting deceit

**DOI:** 10.1371/journal.pone.0215000

**Published:** 2019-04-12

**Authors:** Sophie van der Zee, Ronald Poppe, Paul J. Taylor, Ross Anderson

**Affiliations:** 1 Department of Applied Economics, Erasmus School of Economics, Erasmus University Rotterdam, Rotterdam, The Netherlands; 2 Computer Laboratory, University of Cambridge, Cambridge, United Kingdom; 3 Information and Computing Sciences, Utrecht University, Utrecht, The Netherlands; 4 Psychology, Lancaster University, Lancaster, United Kingdom; 5 Psychology, University of Twente, Enschede, The Netherlands; Arizona State University, UNITED STATES

## Abstract

We present a new signal for detecting deception: full body motion. Previous work on detecting deception from body movement has relied either on human judges or on specific gestures (such as fidgeting or gaze aversion) that are coded by humans. While this research has helped to build the foundation of the field, results are often characterized by inconsistent and contradictory findings, with small-stakes lies under lab conditions detected at rates little better than guessing. We examine whether a full body motion capture suit, which records the position, velocity, and orientation of 23 points in the subject’s body, could yield a better signal of deception. Interviewees of South Asian (*n* = 60) or White British culture (*n* = 30) were required to either tell the truth or lie about two experienced tasks while being interviewed by somebody from their own (*n* = 60) or different culture (*n* = 30). We discovered that full body motion–the sum of joint displacements–was indicative of lying 74.4% of the time. Further analyses indicated that including individual limb data in our full body motion measurements can increase its discriminatory power to 82.2%. Furthermore, movement was guilt- and penitential-related, and occurred independently of anxiety, cognitive load, and cultural background. It appears that full body motion can be an objective nonverbal indicator of deceit, showing that lying does not cause people to freeze.

## Introduction

Although nonverbal cues to deception have been studied for decades, the current literature is characterized by inconsistent and often contradictory findings, leading many researchers to focus their research on verbal cues [1]. For example, both leg movements and head movements have been found to both decrease [2, 3] and increase [4, 5] when lying. In an effort to clarify these mixed results, a number of researchers have provided meta-analyses [6, 7, 8, 9]. These concluded that the majority of cues (about 75%) that were related to deceit as measured in deception experiments, were not actually related to deceit (e.g. gaze aversion and postural shifts). For the correlations that appeared to be stable, the relationship between the cue and lying was typically weak [7, 10]. For example, DePaulo et al. [7] found that amongst nonverbal cues, only illustrators (movements that accompany speech; *d* = -.14), general fidgeting (*d* = .16), and chin raising (*d* = .25) were significantly related to deception. In practice, this means that real-life differences between truth tellers and liars are less clear than is stated in some police interview manuals and accounts to the general public [5, 7].

Researchers have therefore sought to identify moderators of cue saliency. Zuckerman et al. [6] argued that the type and magnitude of deceptive behavior is dependent on three factors: the extent to which liars experience arousal and emotions such as guilt, fear, and delight [11]; the extent to which they experience cognitive load as a result of difficulties constructing and maintaining the lie [6, 10]; and how able they are to control their ‘lying behavior’ [12]. Each of these three factors has been found to influence a liar’s behavior in different and sometimes contradictory ways [6, 9, 13]. Emotions like guilt and fear have been found to decrease the production of illustrator gestures [14], while the increased physiological arousal caused by fear may increase self-adaptors and fidgeting [6]. Similarly, compared to truth telling, the excitement experienced when lying can increase the occurrence of body movements like smiling and illustrators [9], while cognitive load can reduce behaviors such as hand movement [15], foot and leg movement [8], overall body animation [9], and eye blinks [16]. Finally, attempting to control one’s ‘lying behavior’ has been shown to reduce certain types of behavior, leading to a rehearsed and rigid movement pattern [17]. For example, the suppression of a specific facial expression led to the reduction of all facial expressions [18]. As a consequence, either an increase or a decrease in specific behaviors can be a sign of lying (e.g., an increase in fidgeting caused by lie-related nervousness or a decrease due to increased cognitive load or attempted behavioral control). Clearly, examining the effects of such moderators is important if research is to understand nonverbal cues to deceit.

While researchers have gone to great lengths to increase the salience of cues within their studies, comparatively little effort has been made to improve the sensitivity with which nonverbal behavior is measured. As with most signal detection problems, effective progress within the field is made by both reducing the ‘noise’ surrounding the signal (i.e., by increasing its salience within the context) and by improving the efficiency with which the signal itself is measured [19]. So far, most nonverbal deception research has derived its data by having researchers manually code videos, typically using a classification scheme [20]. Although these studies have provided valuable insights, there may be room for improvement because manual coding is associated with several problems. First, manual coding requires the researcher to decide beforehand what cues to code. This top-down research approach can be useful, but the majority of studied cues are unrelated to deceit [8] and it can curtail the detection of novel and lesser-known cues. This is arguably why recent studies using post hoc cue selection have had success in discovering new, unexplored cues [21, 22, 23]. Second, because manual coding is time-consuming, it creates a trade-off between the amount of data collected and the number of coded actions [20]. In other words, there is a limit to the diversity of behavior a research team can practically code, which again limits the chances of finding cues that are related to deceit. Third, manual coding is subjective and can cause reliability issues [24] that can lead to both false alarms and missed positives (i.e., cues going undetected). Using multiple coders and then calculating an inter-rater reliability score can help reduce this subjectivity issue but it does not fully solve it. Fourth, manual coding in deception research is often expressed binomially (e.g., head movement: yes or no) and, only on rare occasions, includes the duration of a movement [25]. The magnitude and direction of the movement are typically not taken into account, despite evidence that such differences carry the ‘meaning’ of the movement [26]. Fifth, researchers usually focus their coding on large movements, so small movements may go undetected.

All five of these issues may be tackled by replacing manual coding with an automatic measurement of nonverbal behavior. This can be done in many ways such as the automatic coding of video footage [27] or the analysis of recorded motion capture data [20, 23, 28]. Automatic coding of video data does not require interpretation and is therefore more objective than manual coding. However, automatic video coding is typically based on 2D representations of behaviors that are 3D in real life and this has been shown to impair the resulting analysis [27]. Additionally, video quality issues can significantly impair the robustness of automatic video-based analyses [29].

Another alternative to manual coding is the use of full body motion capture systems that deliver rich, 3D data of bodily movements. For example, an Xsens MVN full body suit contains 17 inertial sensors that register movement up to 120 times per second in three dimensions for 23 joints. Although one inertial sensor is placed on the head, allowing for the registration and analysis of head movement, the Xsens system does not capture facial expressions. The suit registers both local and global position data so the experimenter knows how the subject’s limbs move with respect to each other and to the floor. With this information it is possible to generate a 3D representation of the subject. Importantly, automated measurement methods like motion capture suits are typically quantitative. Because there is no human in the analysis loop, the measurement is objective. There is also no interpretation of the data, which means it is less likely that cues are missed or misidentified. Taking advantage of these methodological aspects, motion capture equipment is increasingly being used in a wide variety of research fields, including diagnosing post-traumatic stress disorder (PTSD); Scherer et al. [30] have shown that a Kinect, a depth camera allowing for remote motion capture, can be used to measure behaviors that are indicative of PTSD, such as rhythmic fidgeting and rocking.

Early results from automatic analyses of nonverbal behavior to detect deceit are promising. Using a video-based automatic analysis of deceptive facial expressions, Bartlett et al. [31] were able to identify deceit with 85% accuracy, while humans in their experiment did not perform better than 55%. This study demonstrates that some behaviors indicative of deception are difficult to pick up for humans, but can be robustly identified using automatic analyses. Recently, Wu et al. [32] took a multi-modal approach and demonstrated that detection rates increase when using complementary information from the face, the voice, and linguistics. Similarly, Meservy et al. [29] were able to correctly identify deceit with 71% accuracy using a neural network with input from facial expressions and gestures; and analyses of hand and face movement have been used to automatically classify deception-related behaviors such as agitation and behavioral control [33]. Although these studies provide an objective measure of specific types of deceptive behavior, they are often limited to examining facial expressions [31, 32] or specific body parts such as the face and hands [29, 33]. This is a limitation because several manually coded studies have found that other aspects of body movement such as foot, leg, and head movements may also be indicative of deception [2, 3, 4, 5] Accuracy can further be improved when multiple cues (e.g., cue clusters) are considered [9, 34]. Recent evidence of this comes from Duran et al. [28], who used motion capture equipment to measure body and facial movements. They found that participants generally moved less when lying. Given that participants voluntarily lied or not, the direction of the causality of their behavior and their choice to lie is unknown. In the current paper, we investigate whether these results generalize to the common situation of a seated interview in which participants can prepare their lies. We further research the effect of cognitive load and emotion, both of which are known moderators of nonverbal behavior, on the delivery of the lie.

### Current study

To take an inclusive approach to investigating nonverbal indicators to deceit, in the current study we chose to implement an automatic analysis based on motion capture data because it allows for an analysis of subtle full body motions. A sensitive analysis is more effective if there is no systematic variance in the data. One factor that may cause such a bias is the cultural background of participants. Although no culture-specific nonverbal cues to deceit have been identified so far, there is evidence that cultural background can affect people’s interpersonal behavior [35] and their verbal behavior when lying and telling the truth [36]. For example, even when being truthful, Surinamese participants naturally showed more nonverbal behaviors that are related to deception compared to Dutch participants [37]. These potential differences in baseline behavior between people of different cultures led us to include cultural background as an independent variable in this study.

To examine the impact of lying on nonverbal behavior, we conducted an experiment in which we compared full body behavior of interviewees telling truths or lies. The interview comprised of two tasks to investigate whether interview techniques that have previously shown to magnify behavioral differences between truth tellers and liars [5] have a similar enhancing effect on full body movement. We measured full body movement using Xsens MVN motion capture suits. To achieve a culture-sensitive analysis of lying behavior, we compared the behavior of interviewees with a low-context cultural background (i.e., from a predominantly individualistic society) with interviewees with a high-context cultural background (i.e., from a predominantly collectivistic society) [38, 39]. We did so in both within-cultural and cross-cultural interviews. Because theoretical models (i.e., the emotional, cognitive load, and attempted behavioral control approaches) [6] and empirical research have demonstrated that movement can both increase and decrease when lying [2, 3, 4, 5], we refrained from postulating directive hypotheses.

## Methods

This experiment was approved by the Lancaster University Research Ethics Committee, and is in line with the World Medical Association Declaration of Helsinki.

### Participants

One hundred-and-eighty students and employees from Lancaster University (*M* Age = 22.43 years, Range 18–84, Males = 80) volunteered to participate as either an ‘interviewee’ or ‘interviewer.’ The dataset comprised of 18 male pairs, 28 female pairs, and 44 mixed pairs. The experiment took approximately 70 minutes and both interviewees (*n* = 90) and interviewers (*n* = 90) were paid £7.50 for their participation.

### Design

A 2 (Veracity) x 2 (Culture) x 2 (Task) mixed design was implemented, with task as a within subjects variable. Half of the interviewees (*n* = 45) were instructed to respond truthfully to the questions about the two tasks and half were instructed to lie. Participants were divided in low-context and high-context communicators based on their self-reported country of birth [38]. We combined them in three kinds of interviewer-interviewee pairs: British interviewer and interviewee (30 pairs; within-culture); South Asian interviewer and interviewee (30 pairs; within-culture); and British interviewer and South Asian interviewee (30 pairs; between-culture). The latter cross-cultural condition was included because the nature of interactions between low-context interviewers and high-context suspects is relevant for law enforcement practice in predominantly low-context countries such as the UK and the US.

### Measuring absolute movement

**Fig 1:**
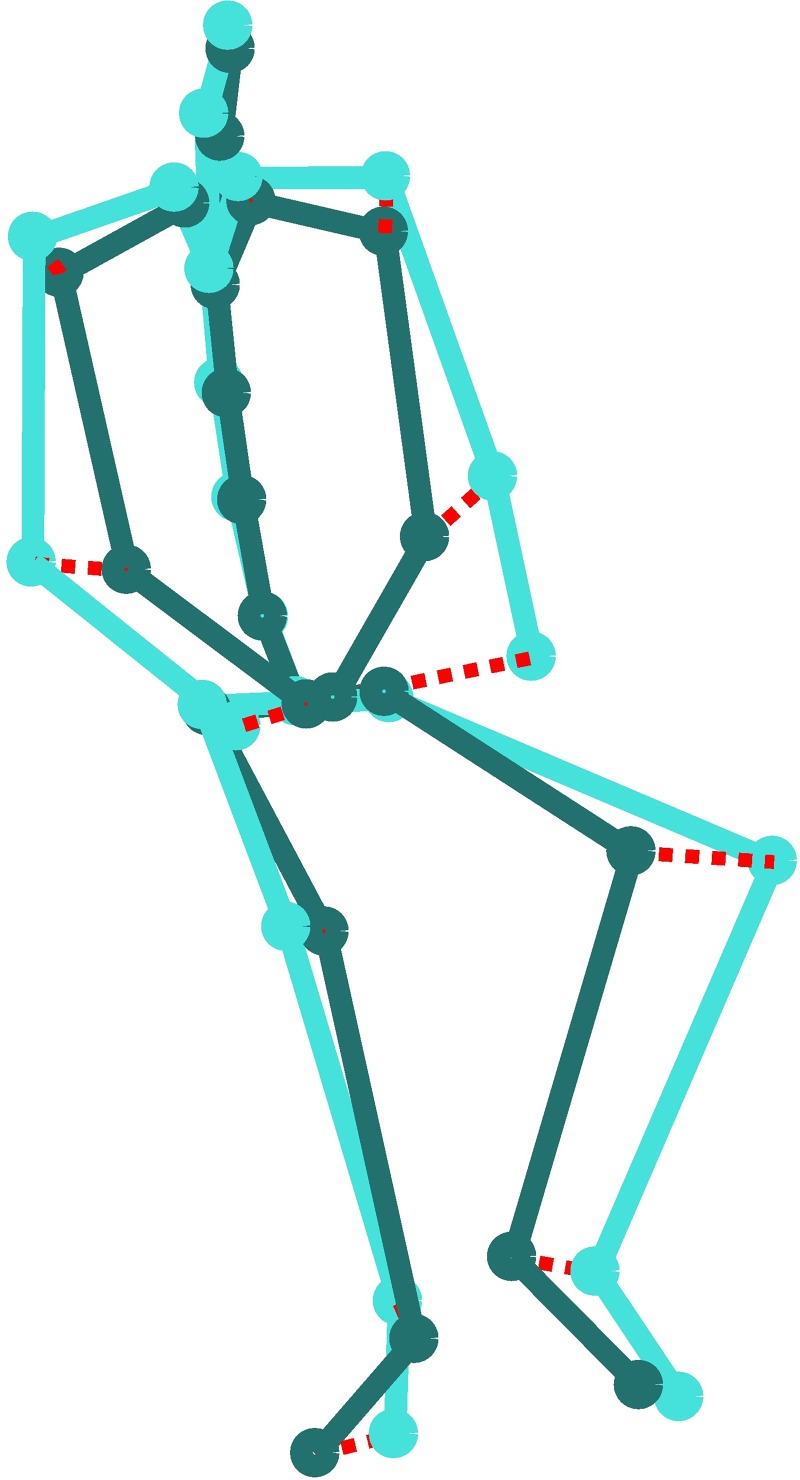
Illustration of absolute measure for full body motion. Two poses in shades of blue, with the distance between pairs of joints indicated by dashed red lines.

Absolute movement was measured using two full body Xsens MVN motion capture suits. For each person, we obtained the 3D positions of 23 joints in the body, which we normalized for global position in space using the processing described in [20]. The distance between poses of subsequent frames was then calculated as the sum of the differences of all joints. Absolute movement was measured as the mean value of the differences between pairs of subsequent poses over time. To calculate full body movement, we took a participant’s normalized body pose at a certain point in time (time frame *t*) and compared it to his or her pose at the next time frame *t+1*. If the poses exactly overlapped, no movement had taken place, resulting in an absolute movement score of 0. If the poses differed between the two time frames, we calculated the pose difference in centimeters for each joint and summed the differences. This results in a full body absolute movement score for the selected start frame. Fig 1 shows a visual representation of this method. Next, we repeated these calculations for all time frames over the duration of an interaction. This resulted in a full body movement score that represents how many centimeters per second a participant moves with his entire body.

To calculate absolute movement of specific body parts (i.e., arms, legs, head, and body), we took into account only the differences for the subset of relevant joints. For example, to determine absolute arm movement, movement data from hand, wrist, lower arm, and upper arm were included but not data from head or leg movements. Before calculating the absolute movement per body part, we aligned the subset of joints on the body part root (i.e., shoulder, hip, neck, or pelvis, respectively). This effectively eliminates the movement due to movement in other parts of the body. For example, leaning forward affects the shoulder locations. By aligning on the shoulder, solely the movement of the (upper and lower) arm can be measured.

### Materials

#### Post-interview questionnaire

On completing the interview, interviewers and interviewees completed a post-interview questionnaire that required them to respond to a series of statements using a Likert scale ranging from ‘not at all’ (1) to ‘very much’ (7). The statements comprised a measure of cultural background and stereotype threat. They also asked participants to indicate how difficult they found their assignment as an indication of experienced cognitive load and how they felt after the interview on a range of emotions (i.e., frightened, anxious, fearful, nervous, guilty, regretful, repentant, penitential, happy, cheerful, pleased, and enthusiastic).

#### Cultural background

To ensure the communication preferences of participants is consistent with our high-/low-context assignment based on country of birth, participants completed a 22-item cultural scale [38]. The 22-items captured participants attitudes towards indirect communication (3 items, e.g., “I catch on to what others mean even when they do not say it directly”), sensitivity for maintaining social harmony (5 items, e.g., “I often bend the truth if the truth would hurt someone”), humbleness in communication (8 items, e.g., “I am modest when I communicate with others”), and persuasion and multitasking (6 items, e.g., “I do not like to engage in several activities at the same time). One item in the original scale (Humbleness: “I listen very carefully to people when they talk”) was excluded from analysis because it had an unduly detrimental impact on the scale’s internal consistency (22 items, α = .65). The remaining 21-item scale showed acceptable consistency (α = .71).

#### Stereotype threat

To better understand the impact of cultural background on interviewees’ experiences and feelings, especially when interacting cross-culturally, participants completed a 4-item stereotype threat measure. Stereotype threat is a situational predicament in which one can feel at risk of confirming negative stereotypes others may hold on their social group and experiencing this threat can cause behavioral changes [40]. The 4-item measure asked participants: i) People sometimes make judgments about my honesty based on my ethnic group; ii) People sometimes make judgments about my trustworthiness based upon my ethnic group; iii) People sometimes think I am not a truthful person based on my ethnic group; and, iv) People sometimes think my behavior is suspicious based on my ethnic group. The internal consistency of the stereotype threat measure on our data was high (α = .93).

### Procedure

The experiment comprised a pre-interview and an interview stage. The pre-interview stage required interviewees to complete two tasks (i.e., playing a computer game and handling a missing £5 note), while the interviewer received instructions about the interview. On completion of these tasks, the interviewee and interviewer were led to the interview room where they were each fitted with a motion capture suit. During the interview, interviewers read a scripted set of questions out loud. Half of the interviewees were instructed at the beginning of the experiment that they were to respond truthfully to the questions of the interviewer, while the other half were instructed to lie. Interviewees, regardless of veracity condition, were told that their name would be put in a prize draw for £50 if they managed to convince the interviewer that they were being truthful about both topics. This incentive was implemented to increase the stakes and to encourage participant motivation. In reality, to ensure equal treatment, all interviewees’ names were put in the prize draw.

#### Pre-interview

After giving informed consent, interviewees were told that they were about to complete two tasks and that they would subsequently be interviewed about those tasks by another participant. Interviewees remained unaware of the content of the interview questions until the start of the interview. Next, they received instructions about the two pre-interview tasks. These instructions differed depending on veracity condition. The first task required participants to play a computer game called ‘Never End’ for seven minutes. ‘Never End’ is a strategic game in 2D that can be played online for free (available at https://www.freeonlinegames.com/game/never-end). The objective of the game is to collect keys and open doors that lead to new rooms. Each room is a maze and the player can walk, jump, and rotate the entire room with 90 degrees to achieve this goal. The more keys are collected and the more doors are opened, the higher the score. In this game, the order of events is critical, because obstacles and spikes may kill the character if actions are performed in the wrong order. Interviewees in the truth condition played the game for seven minutes, while interviewees in the lie condition did not play the game. Instead, they received an information sheet about the game that provided them with details that enabled them to fabricate a story about playing the game. They had seven minutes to study this information sheet and prepare their lie. This design enabled interviewees in both conditions to describe how they played the computer game, although only the participants in the truth condition actually had the experience of doing so.

The second task involved handling a lost wallet that contained a £5 note. In the truth condition, participants were asked to bring the wallet to the lost-and-found box while, in the lie condition, participants were asked to remove the £5 note from the wallet and hide it somewhere on their body. These participants were instructed to put the wallet back where they found it and fabricate a story about bringing the wallet to lost and found. During the interview, interviewees in the lie condition were instructed to hide the fact that they had stolen the £5 note and to pretend that they brought the wallet to the lost-and-found box.

#### Interview

After 12 minutes, the experimenter returned to the lab and checked that the interviewee had followed the instructions correctly. She then removed all remaining evidence (e.g., the wallet in the lie condition) and invited the interviewer into the room. She helped both interviewer and interviewee into Xsens MVN motion capture suits and invited them to sit on one of two chairs that were positioned facing one another. To ensure participants had an unobstructed view of the other’s behavior, no table was situated between them.

Interviewers had previously been informed (while the interviewee was carrying out the pre-interview tasks) that they were to ask a set of pre-made questions about the computer game ‘Never End’ and about a missing £5 note. The questions about the missing £5 note were asked in normal order while the questions about the computer game of ‘Never End’ required interviewees to recall the event in reverse order. The latter was done to increase the cognitive load experienced by liars, which had previously been shown to magnify behavioral differences between truth tellers and liars [5]. Reverse order questions about the game followed the format used in previous research, with questions incrementally moving back through the experience of interest and asking for specific details at each stage [41]. While this is not the only way to implement the reverse order technique (i.e., others ask for a reversed free recall), this approach had the advantage in this study of allowing us to standardize across conditions and the range of information discussed by interviewees. The reverse order questions were: (1) Please tell me how your game ended; (2) At what level did your game end? (3) What was your total score? (4) How was the score calculated? (5) For what item did you get the most points? (6) What happened when you went through an exit? (7) How many times did your character die? (8) How did your character usually die? (9) Please tell me about the lay-out of the game: any specific colors, effects or sounds? (10) Please tell me about the commands; (11) What is the main aim of this game? (12) Please tell me how your game started; and (13) Please tell me how you felt when playing the game. Normal order questions about the missing £5 note were: (1) Did you take the £5 while you were here playing ‘Never End’? (2) Please explain what you were doing while you were in this room from start to finish. Include all details please; (3) So this means you went out of the room? (4) How long was the walk to the room where the lost property box was located? (5) Did you see anyone in the hallway while you were walking to the lost property box? (6) If so, how did he/she look like? (7) When you arrived in the room, how many items were in the lost property box? (8) Could you describe these items for me please? (9) What was written on the box? (10) What was next to the box? (11) Describe the room the lost property box was in; (12) Where did you put the wallet in the box, in relation to the other items? (13) How long were you gone from this room? (14) How do you feel about this money gone missing? and (15) Lastly, I will ask you again: did you take the £5?

Interviewers were instructed that their task was to decide, for each topic, whether or not they thought the interviewee was being truthful. They were told the interviewee may be truthful about both topics, deceptive about both topics, or be truthful about one topic and deceptive about the other topic. To provide an incentive, interviewers were told that if their judgments were correct, their name would be put in a prize draw for £50. In reality, to ensure equal treatment, all interviewers’ names were put in the prize draw. After setting up the equipment, the experimenter handed the interviewer his or her first set of questions and then retreated to monitor the incoming data. The participants spoke for 2.5 minutes about the computer game ‘Never End’, followed by 2.5 minutes about the missing £5 note. Interviews were cut off after 2.5 minutes regardless of how many questions were asked in order to keep the length of the interactions consistent.

## Results

### Cultural background check

The 21-item cultural scale provided the opportunity to compare the culture-specific communication preferences and beliefs of participants to their self-declared ethnicity [38]. An analysis of the average response over the 21 items revealed that participants classified as high-context scored higher on this scale (*M* = 5.06, *SD* = .56) than participants classified as low-context (*M* = 4.85, *SD* = .52), *t*(178) = -2.61, *p* = .010, suggesting that the initial division based on country of birth was acceptable. To reinforce this assessment, we examined participants’ average stereotype threat score as a function of their assigned culture, since those from high-context cultures typically report feeling greater stereotype threat than those from low-context cultures [42]. Participants who were classified as high-context (*M* = 3.33, *SD* = 1.73) reported experiencing more stereotype threat than participants who were classified as low-context (*M* = 1.88, *SD* = 1.02), *t*(178) = -6.84, *p* < .001. A follow-up Cultural background x Veracity ANOVA on average stereotype threat score indicates that stereotype threat perceptions were not moderated by veracity condition, *F*(1, 176) = 3.80, *p* = .053, η^2^_p_ = .02. Taken together, these results demonstrate that the South Asian participants in our study are more collectivistic and experience much higher stereotype threat that the British participants, providing support for our cultural division based on self-reported country of birth.

### Emotion check

**Fig 2:**
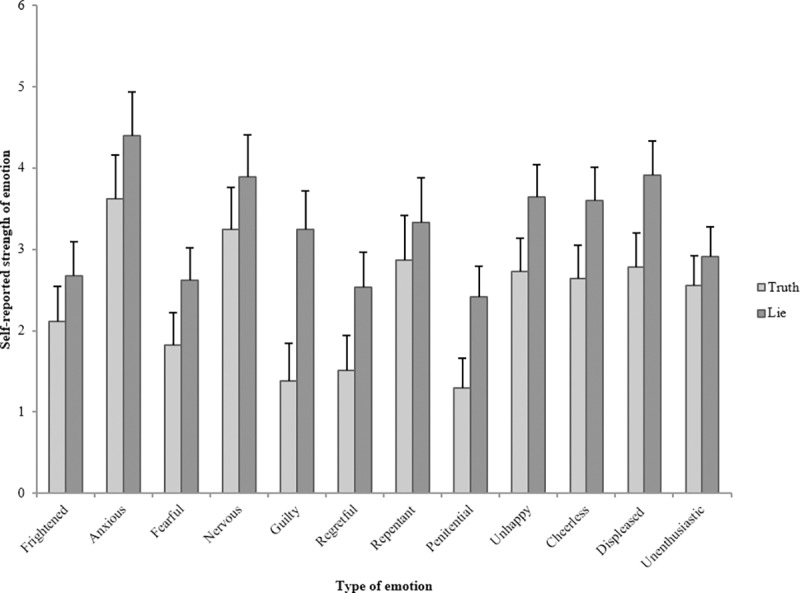
The effect of veracity on a range of self-reported emotions. Error bars = 95% CI.

To examine the relationship between cultural group and participants’ self-reported emotional experiences, we conducted one 2 (Veracity condition: truth and lie) x 3 (Culture condition: low-context, high-context, and mixed) MANOVA with reported feelings of being Frightened, Anxious, Fearful, Nervous, Guilty, Regretful, Repentant, Penitential, Happy, Cheerful, Pleased, and Enthusiastic as the dependent variables. We have reverse-scored the positive emotions (Happy–Unhappy, Cheerful–Cheerless, Pleased–Displeased, and Enthusiastic–Unenthusiastic) in order for all emotions to be scored in the same direction (i.e., the higher the more negative). Interviewees’ emotional experience varied as a function of both Veracity, *F*(12, 73) = 3.81, *p* < .001, η^2^_p_ = .39, and Culture, *F*(24, 148) = 1.65, *p* = .038, η^2^_p_ = .21. Fig 2 illustrates the effect of Veracity on self-reported emotions. As can be seen from Fig 2, compared to participants who told the truth, participants who lied reported feeling more Anxious, *F*(1, 184) = 4.16, *p* = .045, η^2^_p_ = .05, more Fearful, *F*(1, 84) = 8.09, *p* = .006, η^2^_p_ = .09, more Guilty, *F*(1, 84) = 31.18, *p* < .001, η^2^_p_ = .27, more Regretful, *F*(1, 84) = 10.96, *p* = .001, η^2^_p_ = .12, more Penitential, *F*(1, 84) = 18.12, *p* < .001, η^2^_p_ = .18, more Unhappy, *F*(1, 84) = 10.21, *p* = .002, η^2^_p_ = .11, more Cheerless, *F*(1, 84) = 10.91, *p* = .001, η^2^_p_ = .12, and more Displeased, *F*(1, 84) = 14.39, *p* < .001, η^2^_p_ = .15. There were no differences across Veracity for feeling Frightened, *F*(1, 84) = 3.29, *p* = .073, η^2^_p_ = .04, feeling Nervous, *F*(1, 84) = 3.03, *p* = .085, η^2^_p_ = .04, feeling Repentant, *F*(1, 84) = 1.45, *p* = .233, η^2^_p_ = .02, and feeling Unenthusiastic, *F*(1, 84) = 1.83, *p* = .180, η^2^_p_ = .02.

**Fig 3:**
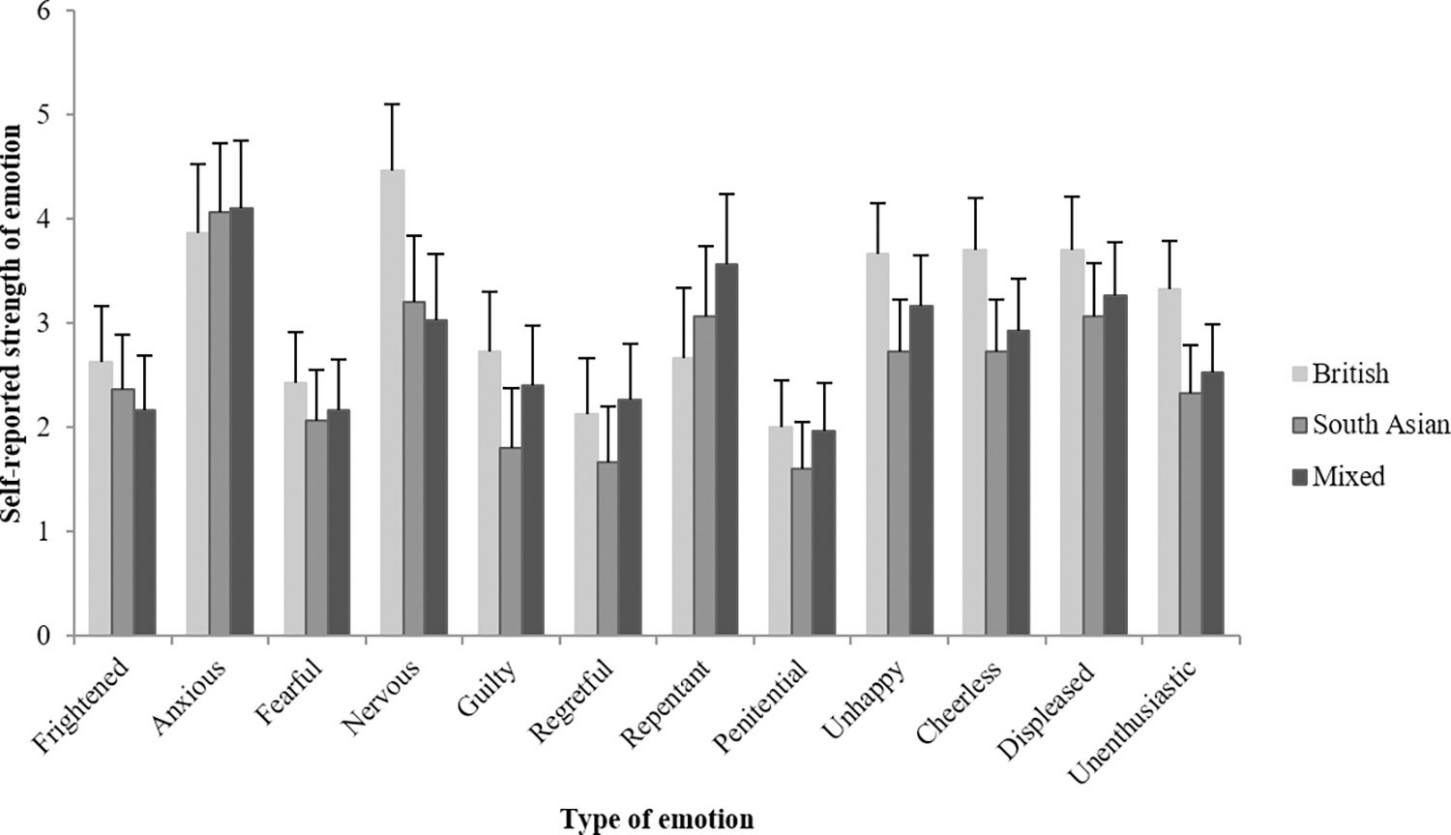
The effect of culture on a range of self-reported emotions. Error bars = 95% CI.

Fig 3 illustrates the direction of the significant effects that Culture has on Emotion experience when telling truths or lies. Since this study comprises three cultural conditions (low context, high context, and mixed), Bonferroni corrections are applied to the post-hoc testing of culture effects. Culture condition affected feelings of Nervousness, *F*(2, 84) = 5.98, *p* = .004, η^2^_p_ = .13, with interviewees in the low-context condition (*M* = 4.47, *SD* = 1.94) feeling more nervous than high-context interviewees in both the high-context (*M* = 3.20, *SD* = 1.92), *p* = .019 and mixed condition (*M* = 3.03, *SD* = 1.73), *p* = .007; feelings of Unhappiness, *F*(2, 84) = 3.58, *p* = .032, η^2^_p_ = .08, with interviewees in the low-context condition (*M* = 3.67, *SD* = 1.49) reporting feeling unhappier than interviewees in the high-context condition (*M* = 2.73, *SD* = 1.39), *p* = .027; feelings of Cheerlessness, *F*(2, 84) = 4.15, *p* = .019, η^2^_p_ = .09, with interviewees in the low-context condition (*M* = 3.70, *SD* = 1.54) reporting feeling more cheerless than interviewees in the high-context condition (*M* = 2.73, *SD* = 1.31), *p* = .023, and feeling Unenthusiastic, *F*(2, 84) = 5.39, *p* = .006, η^2^_p_ = .11, with interviewees in the low-context condition (*M* = 3.33, *SD* = 1.21) reporting feeling more unenthusiastic than interviewees in the high-context condition (*M* = 2.33, *SD* = 1.24), *p* = .008 and the mixed condition (*M* = 2.53, *SD* = 1.28), *p* = .045. Culture condition did not affect feeling Frightened, *F*(2, 84) = .78, *p* = .462, η^2^_p_ = .02, feeling Anxious, *F*(2, 184) = .15, *p* = .865, η^2^_p_ < .01, feeling Fearful, *F*(2, 84) = .61, *p* = .548, η^2^_p_ = .01, feeling Guilty, *F*(2, 84) = 2.67, *p* = .075, η^2^_p_ = .06, feeling Regretful, *F*(2, 84) = 1.39, *p* = .255, η^2^_p_ = .03, feeling Repentant, *F*(2, 84) = 1.80, *p* = .172, η^2^_p_ = .04, feeling Penitential, *F*(2, 84) = .93, *p* = .400, η^2^_p_ = .02, and feeling Displeased, *F*(2, 84) = 1.57, *p* = .215, η^2^_p_ = .04.

### Full body motion

**Fig 4:**
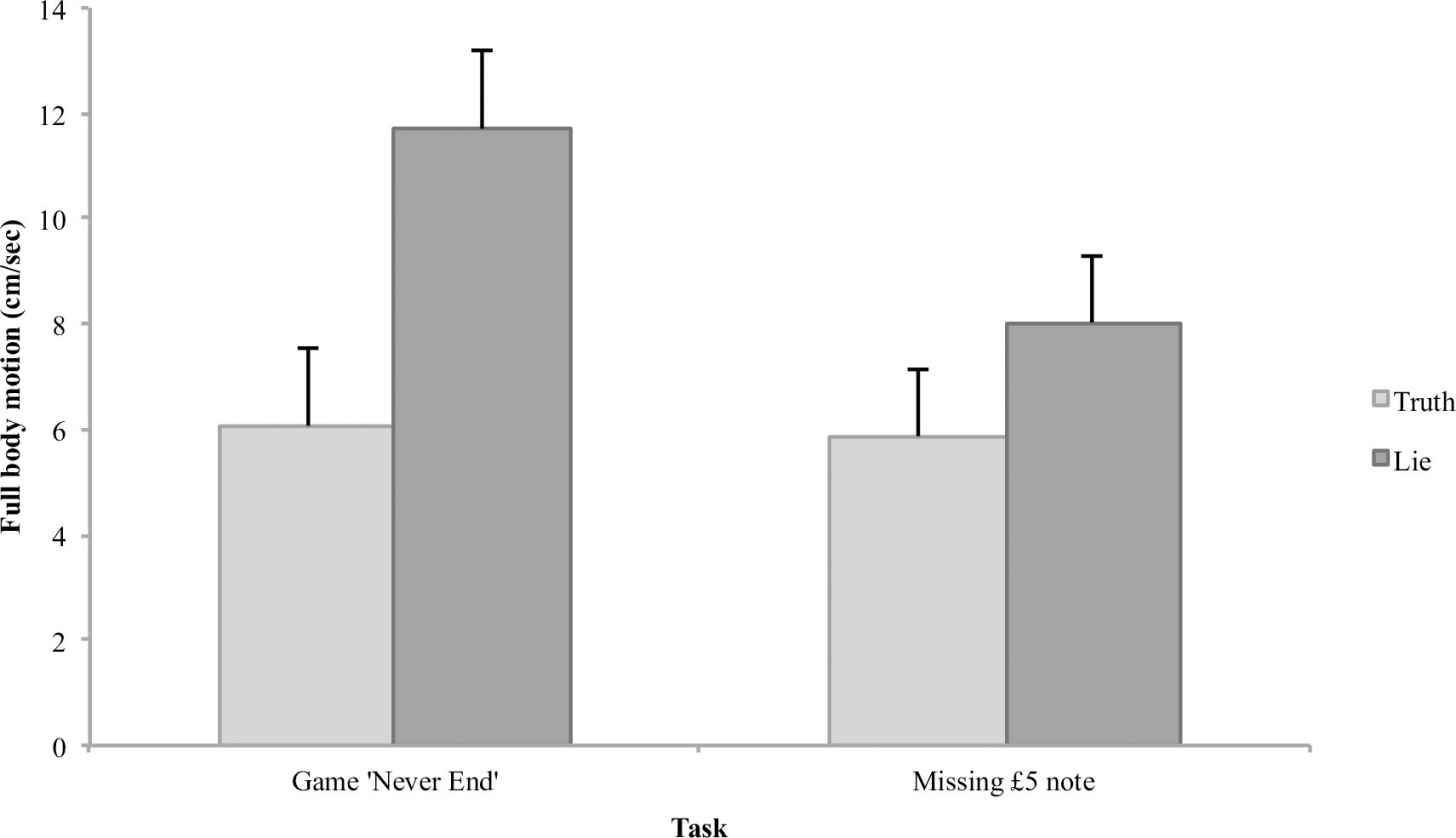
The effect of veracity and task on full body motion in cm/sec. Error bars = 95% CI.

To examine whether truth tellers and liars show different nonverbal movement and to test whether or not this movement was moderated by cultural context, we examined absolute movement (i.e., displayed as centimeters per second) as a function of Veracity condition and Culture condition. Fig 4 shows the full body motion data as a function of Veracity and Task across Culture conditions. A 2 (Veracity condition) x 3 (Culture condition) x 2 (Task) mixed ANOVA with Task as the repeated measure and full body movement as the dependent variable revealed main effects for both Task, *F*(1, 84) = 36.66, *p* < .001, η^2^_p_ = .30, and Veracity condition, *F*(1, 84) = 17.99, *p* < .001, η^2^_p_ = .18, which were subsumed in a Task x Veracity interaction, *F*(1, 84) = 29.41, *p* < .001, η^2^_p_ = .26. Although in general liars (*M* = 9.87, *SD* = 5.32) moved more than truth tellers (*M* = 5.97, *SD* = 3.67), how much more interviewees moved was dependent on what task they were discussing. While truth tellers moved similar amounts during both the computer game ‘Never End’ task (*M* = 6.07, *SD* = 3.54) and the missing £5 note task (*M* = 5.87, *SD* = 3.81), liars moved much more when being interviewed about the computer game ‘Never End’ (*M* = 11.70, *SD* = 5.95) compared to the missing £5 note (*M* = 8.03, *SD* = 4.69). In order to manipulate cognitive load, interviewees were asked to answer questions about the missing £5 note in forward order, whilst being asked to answer questions about playing the computer game ‘Never End’ in reverse order. As a result, Task magnified the behavioral differences between truth tellers and liars, an effect arguably caused by cognitive load inducing interviewing techniques. Importantly, Culture did not affect full body movement, *F*(2, 84) = .50, *p* = .609, η^2^_p_ = .01.

When examining the movement data in more detail, we found that the full body movement result (i.e., an interaction effect of Task and Veracity condition) was replicated at the level of individual limbs. We ran a series of six equivalent mixed ANOVAs with Veracity and Culture as the independent variables and Task as the repeated measure. We set alpha to 5% for all tests in this paper. Here, in order to avoid Type 1 errors due to multiple testing, we adjusted alpha to .05 / 6 = .008. These tests revealed significant interaction effects between Task and Veracity for the left arm, *F*(1, 84) = 9.46, *p* = .003, η^2^_p_ = .10, right arm, *F*(1, 84) = 21.78, *p* < .001, η^2^_p_ = .21, right leg, *F*(1, 84) = 9.68, *p* = .003, η^2^_p_ = .10, head *F*(1, 84) = 21.83, *p* < .001, η^2^_p_ = .21, and torso, *F*(1, 84) = 17.48, *p* < .001, η^2^_p_ = .17. Due to the multiple testing alpha correction, the interaction effect of Task and Veracity on movement in the left leg is no longer significant, *F*(1, 84) = 6.47, *p* = .013, η^2^_p_ = .07. When relying on the adjusted alpha, Culture did not affect movement in any of limbs.

### Detecting deception on an individual level

To measure how discriminative full body movement would be when applied on an individual level, we calculated how much truthful interviewees moved in total when discussing the game ‘Never End’ (*M* = 6.07, *SD* = 3.54) and the missing £5 note (*M* = 5.87, *SD* = 3.81), and how much deceptive interviewees moved in total when discussing the game ‘Never End’ (*M* = 11.70, *SD* = 5.95) and the missing £5 note (*M* = 8.03, *SD* = 4.69). Subsequently, we ran a binary logistic regression with two predictors to calculate the predictive value of full body movement for deception detection purposes. The first predictor concerns full body movement when discussing the game ‘Never End’ and the second predictor concerns full body movement when discussing the missing £5 note. A test of the full model against a constant-only model was statistically significant, indicating that the full body movement predictors reliably distinguished between truth tellers and liars, *X*^2^ (2) = 35.19, *p* < .001, Nagelkerke *R*^2^ = .43. Overall, we correctly classified 74.4% (truths: 80.0%, lies: 68.9%) of the interviewees as either being truthful or deceptive based on one aggregated full body movement measure. We ran a second binary logistic regression to calculate whether a model based on individual limb movement instead of one aggregated full body measure could lead to a higher predictive validity. We included absolute movement values of both arms, both legs, the head, and the body during the interview about the game ‘Never End’ and during the interview about the missing £5 note (12 predictors) to predict if the participant was lying or being truthful. Again, a test of the full model against a constant-only model was statistically significant, indicating that the individual limb movement predictors, as a set, reliably distinguished between truth tellers and liars, *X*^2^ (12) = 48.45, *p* < .001, Nagelkerke *R*^2^ = .56. Overall, we correctly classified 82.2% (truths: 88.9%, lies: 75.6%) of the interviewees as either being truthful or deceptive based on the combined movement in their limbs.

### Influence of cognitive load and emotion on body motion

To measure whether self-reported difficulty, implemented as a measure of experienced cognitive load, affects movement, we calculated correlations between difficulty and the interviewee’s full body movement when answering questions about the game ‘Never End’ and when answering questions about the missing £5 note. Although liars (*M* = 3.29, *SD* = 1.65) did report finding their assignment more difficult than truth tellers (*M* = 2.07, *SD* = 1.23), *t*(88) = 3.99, *p* < .001, this increase in difficulty did not affect full body movement when answering questions about the game ‘Never End’, *r* = .089, *n* = 90, *p* = .404, nor when answering questions about the missing £5 note, *r* = .038, *n* = 90, *p* = .724. To investigate if any specific limbs were affected by cognitive load, we calculated a correlation matrix of self-reported difficulty on absolute movement in individual limbs when being interviewed about both topics. Movement in none of the limbs was correlated with self-reported difficulty during any of the tasks.

To measure whether experienced emotions have an impact on how much people move, we calculated correlations between the twelve self-reported emotions and the interviewee’s full body movement when answering questions about the game ‘Never End’ and when answering questions about the missing £5 note. We controlled the false discovery rate by applying the Benjamini-Hochberg procedure. We set the critical value for a false discovery rate to .25. The results indicated that feeling guilty and feeling penitential were positively correlated with full body movement, but only when answering reverse order questions about the game ‘Never End’. In other words, interviewees that indicated feeling guilty moved more than interviewees who reported feeling less guilty, *r* = .247, *n* = 90, *p* = .019, i = 2, (i/m)Q = .021. Similarly, interviewees that indicated feeling penitential moved more than interviewees who reported feeling less penitential, *r* = .260, *n* = 90, *p* = .013, i = 1, (i/m)Q = .010. None of the other self-reported emotions were correlated with full body movement.

## Discussion

We started this paper by noting that research on nonverbal indicators of deceit has reported inconsistent and even contradictory results [9] and that the identified cues often have a weak relationship with veracity [6, 7, 8, 9]. We set out to investigate whether this lack of reliable nonverbal cues can be remedied by more sensitive measurements. We used full body motion capture suits to automatically capture movement of each body part and compared total body motion when lying with when being truthful. We did not hypothesize a direction of the results because mixed findings have been reported both on a theoretical and on a practical level. With a medium effect size of .26 (Pearson’s *r*), our results indicate that full body motion is a reliable nonverbal indicator of deceit. When measured accurately and objectively, body motion includes not just discrete, large, and easily coded movements, but also the many smaller movements that people make that are usually not included with manual coding. An examination of full body motion showed that people who lied moved more than people who spoke the truth. Based on the aggregated full body motion measure we could correctly classify 74.4% (truths: 80.0%, lies: 68.9%) of all interactions. When including movement in the individual limbs, we could further increase our correct classification to 82.2% (truths: 88.9%, lies: 75.6%). Compared to an average detection rate of around 54% in similar experimental settings when humans attempt to detect deceit [42] 82.2% is a solid improvement.

To date, the findings in the literature regarding the way in which liars move their bodies are mixed. Since the majority of these studies relied on manual coding, our conclusions may be difficult to compare. Previous research using motion capture equipment to identify the movement patterns of liars is scarce. Interestingly, our main result is at odds with Duran et al. [28]. In their reanalysis of the motion capture data collected by Eapen et al. [43], they found participants appeared to move less when lying and this effect only showed in specific body parts. By contrast, our participants moved more when lying and this effect occurred across all body parts. This discrepancy in results between the two studies could be explained in a number of ways. First, the participants in our experiment knew beforehand whether they had to lie or not and were provided with the opportunity to prepare their lies. In contrast, participants in Eapen et al.'s study had to decide whether to lie or not on the spot. The lack of preparation could have caused differences in behavior. Second, participants in our study were seated in an interview-like setting, whilst participants in Eapen et al.’s study were confronted whilst standing.

Third, while Eapen et al. measured body motion in response to a single veracity question, we have considered time intervals of 2.5 minutes during which several follow-up questions where posed. In Eapen et al.’s short time window, several factors other than veracity may have affected their bodily behavior such as surprise, confrontation, and on-the-spot decision-making. Fourth, Eapen et al. asked participants two questions, one baseline question followed by one veracity question. Because participants only decided on the spot whether or not they would lie in response to the veracity question, one would expect not to find any behavioral differences in response to the baseline question. However, they found that participants who decided to lie in response to the veracity question already showed reduced movement during the baseline question. Duran et al. explained this finding through an anticipation effect, which indeed is a plausible explanation. Arguably, at the moment of answering the baseline question participants may not have known that they were required to lie in response to the next veracity question. If that were true, another interpretation of these findings could be that the reduction in movement is more associated with the type of person that chose to lie in this setting than with the act of lying itself. Because both studies differ methodologically in several ways, it is impossible to disentangle which factors exactly cause participants to move less or more when lying. Future research using motion capture equipment to measure lying behavior in several settings is recommended to address this question.

We tested whether cultural background affected lying behavior. Although previous research has demonstrated differences in the interpersonal behavior of those from low-context (i.e., British) and high-context cultures (i.e., South Asian) [35], we did not find any such differences in full body movement. This finding can be explained in several ways. First, Hall [39] differentiated between low- and high-context cultures based on different preferences in communication patterns, with individuals from high-context cultures making more use of contextual cues in social interactions than individuals from low-context cultures. As a consequence, one would expect behavioral differences between the two types of cultures to be most prevalent in verbal rather than nonverbal communication. Indeed, recent papers comparing the verbal behaviors of low- and high context individuals did find cultural differences in their participants’ baseline behavior, with low-context individuals reporting more details than high-context individuals [36, 44]. Second, the majority of nonverbal behavioral differences between low- and high-context cultures that are reported in the literature are types of nonverbal behaviors that cannot be measured when solely relying on motion capture data. For example, [37] found that high-context individuals tend to avert their gaze, smile, and laugh more than low-context individuals regardless of veracity. In order to automatically analyze these types of behaviors, high-resolution video recordings and corresponding software are needed. A third possible explanation is that the participants we tested did not differ enough from a cultural perspective to elicit distinctive behavioral patterns. All participants in this study were students or employees of Lancaster University, which means that while our participants from low-context cultures were born and raised in South Asian countries, they have also spent a significant amount of time in the UK. This explanation is supported by the relatively small difference between the low- and high-context conditions on the cultural scale [38]. Interestingly, the difference between the groups on the stereotype threat scale [42] was much larger, suggesting that, while the communication preferences of high-context individuals may have changed, feelings of how they are perceived by others have not. In sum, our results provide no evidence to support the suggestion of culture-specific cues to deceit.

Currently, the lack of identified reliable nonverbal indicators of deceit is explained in the literature by the moderating function of emotion, cognitive-load, and attempted behavioral-control. To test whether these factors serve as moderators, we asked participants to self-report how difficult they found their assignment and how they were feeling on a range of emotions. Liars reported finding their assignment more difficult than truth tellers, a finding in line with previous research that demonstrated people experience increased cognitive load when lying [45, 46]. Several previous studies demonstrated that increased cognitive load can lead to a reduction in movement [9, 15]. However, self-reported difficulty (implemented as a measure of cognitive load) was not correlated with movement in any of the limbs during either of the tasks in the current study.

This raises the question of why there may be a disconnect between clear nonverbal changes in behavior when lying and clear changes in experience (as self-reported) when lying. It is impossible to say with certainty but our design does allow us to rule out differences across context, since both liars and truth tellers gave accounts of the game and stealing experience. Nor is it the result of cultural differences, since the results remained the same across all participants. Rather it appears to be the case either that nonverbal changes are driven by mechanisms other than cognitive load or that subjective experience is distinct from objective experience.

The first proposition suggests that factors other than cognitive load related emotions are driving the behavioral changes between the two interview topics. A limitation of the current study is that the variables Task and Interviewing technique are confounded. Participants always answered questions about the stolen money in chronological order and always answered questions about the game ‘Never End’ in reverse order. As a result, it is impossible to disentangle the effects of Task and Interviewing technique on our dependent variable absolute movement. The lack of difference in self-reported difficulty between the two tasks suggests that the nature of the tasks may have been more influential in shaping behavior than the type of questioning.

The second proposition suggests that the two interview topics did differ in the amount of cognitive load elicited in participants, even though no difference was found in self-reported difficulty. There are three arguments supporting this proposition. First, we implemented a reverse order questioning technique to make it more difficult for participants to answer questions about the game ‘Never End’ compared to the stolen £5. We did implement an adjusted version of the reverse order questioning technique consisting of multiple specific questions instead of one open question, which may have altered its effect on cognitive load. Second, previous research on reverse order questioning showed this technique is especially difficult for liars and consequently magnifies the behavioral differences between truth tellers and liars [47]. This magnifying effect also occurred in our study, where we found an interaction effect of Veracity and Task on absolute movement. Specifically, the behavioral differences between truth tellers and liars were especially large when discussing the game ‘Never End’ in reverse order. And third, several recent studies have experimentally demonstrated that a dissociation between objective and subjective emotional experiences can occur [48, 49], suggesting that discrepancies between subjective and objective measurements may occur. This topic requires further investigation.

A discrepancy between subjective and objective measurements may also explain our anxiety related results. While liars reported feeling more negative than truth tellers, correlation analyses indicated that anxiety related emotions did not influence nonverbal behavior. This finding is in contrast with previous research demonstrating that anxiety can increase nonverbal behaviors such as self-adaptors and fidgeting [6]. A possible explanation for this discrepancy is that the stakes in our study were too low to affect the emotional and cognitive processes that can be elicited by lying. The lies were low-stake in the sense that participants would not be punished if they failed to convince the interviewer of their honesty. We did try to increase the stakes in several other ways. We implemented a task with criminal intent. Participants in the lie condition had to steal a £5 note and hide this information from the interviewer by providing a false alibi. We also offered interviewees an incentive by providing them with the chance of winning £50 if they managed to convince the interviewer of their innocence. Based on the self-reported emotions, our efforts to increase the stakes seem to have worked. Liars did report experiencing more anxiety related emotions than truth tellers; these self-reported emotions just did not affect behavior. Future research using high-stake lies incorporating more objective measurements of cognitive load and emotional responses is needed to further disentangle these effects.

### Limitations and future research

In this paper, motion capture equipment instead of manual coding was used to measure movement. The rich and objective data that motion capture equipment provides creates opportunities for exploring new research avenues, such as changes in behavior over time [21, 22], clusters of cues [9, 34], and the exploration of new cues [23]. The first results of such studies are promising. However, this change in methodology also has implications for the type of movements that are analyzed and may have consequently affected the outcomes of this study. To investigate whether the same data can lead to different conclusions based on the type of coding used (i.e., differences between manual coding and automatic coding based on motion capture data), more methodological research on this topic should be conducted in the future, for example by comparing the effectiveness and implications of manual vs. automatic coding. This future research could indicate whether our results can be explained by methodological choices or whether the assumption that cognitive load and anxiety related emotions cause liars to behave differently might need to be reconsidered.

A second limitation associated with the use of motion capture suits is the possible hindrance of natural movement. We did what we could to minimize the effect by giving all participants time to get used to the suit by starting the interview with a baseline, neutral conversation. In future research this potential issue can be solved by using depth cameras or a setup with multiple cameras to create a point cloud model of the subject’s body or by using millimeter-wave radar to measure total movement directly. Such techniques lead to two promising future research avenues. First, the use of cameras also enables the measurement of facial expressions and verbal behavior, allowing for multimodal deception detection [32]. Second, using cameras instead of motion capture suits would allow for the unobtrusive surveillance of subjects in a police interview room or other operational interrogation environment [50]. Remotely measuring nonverbal behavior in an accurate and objective manner will further help in bridging the current gap between theory and practice in improving ways to detect deception [51].
